# Molecular characterization of RNase III protein of *Asaia* sp. for developing a robust RNAi-based paratransgensis tool to affect the sexual life-cycle of *Plasmodium* or *Anopheles* fitness

**DOI:** 10.1186/s13071-020-3889-6

**Published:** 2020-01-29

**Authors:** Majid Asgari, Mahdokht Ilbeigikhamsehnejad, Elham Rismani, Navid Dinparast Djadid, Abbasali Raz

**Affiliations:** 1 Malaria and Vector Research Group (MVRG), Biotechnology Research Center (BRC), Pasteur Institute of Iran, Tehran, Iran; 2 Molecular Medicine Department, Pasteur Institute of Iran, Tehran, Iran

**Keywords:** Vector-borne diseases, Paratransgenesis, *Asaia*, RNAi, RNase III

## Abstract

**Background:**

According to scientific recommendations, paratransgenesis is one of the solutions for improving the effectiveness of the Global Malaria Eradication Programme. In paratransgenesis, symbiont microorganisms are used for distorting or blocking the parasite life-cycle, affecting the fitness and longevity of vectors or reducing the vectorial competence. It has been revealed recently that bacteria could be used as potent tools for double stranded RNA production and delivery to insects. Moreover, findings showed that RNase III mutant bacteria are more competent for this aim. *Asaia* spp. have been introduced as potent paratransgenesis candidates for combating malaria and, based on their specific features for this goal, could be considered as effective dsRNA production and delivery tools to *Anopheles* spp. Therefore, we decided to characterize the *rnc* gene and its related protein to provide the basic required information for creating an RNase III mutant *Asaia* bacterium.

**Methods:**

*Asaia* bacteria were isolated from field-collected *Anopheles stephensi* mosquitoes. The *rnc* gene and its surrounding sequences were characterized by rapid amplification of genomic ends. RNase III recombinant protein was expressed in *E. coli* BL21 and biological activity of the purified recombinant protein was assayed. Furthermore, *Asaia* RNaseIII amino acid sequence was analyzed by *in silico* approaches such as homology modeling and docking to determine its structural properties.

**Results:**

In this study, the structure of *rnc* gene and its related operon from *Asaia* sp. was determined. In addition, by performing superimposition and docking with specific substrate, the structural features of *Asaia* RNaseIII protein such as critical residues which are involved and essential for proper folding of active site, binding of magnesium ions and double stranded RNA molecule to protein and cleaving of dsRNA molecules, were determined.

**Conclusions:**

In this study, the basic and essential data for creating an RNase III mutant *Asaia* sp. strain, which is the first step of developing an efficient RNAi-based paratransgenesis tool, were acquired. *Asaia* sp. have been found in different medically-important vectors and these data are potentially very helpful for researchers studying paratransgenesis and vector-borne diseases and are interested in applying the RNAi technology in the field.

## Background

Vector-borne diseases (VBD) have enormous impacts on human life [1–3]. Malaria is one of the most important protozoan infectious diseases, caused in humans by five different members of the genus *Plasmodium*. Based on the World Health Organization (WHO) annual report, there were approximately 219 million clinical malaria cases in 2017 which resulted in almost 0.5 million deaths [4]. The main at-risk groups for malaria disease are children under the age of five years, but it also affects other risk groups such as pregnant women and non-immune adults [5].

After the failure of Global Malaria Eradication program [6], the WHO has been updating the malaria policy instruments beginning with a consultative review process involving the scientific recommendations and findings of scientists in 2007 [7]. Drug, vaccine, vector control, modeling, M&E and surveillance, integration strategies, health systems and operational research and diagnostics and young investigators/basic research consultative groups were considered. Three main subjects were recommended by the vector control consultative group: designing new and more efficient insecticides, developing of transgenic insects and applying paratransgenesis [8].

Paratransgenesis is a kind of “Trojan horse” for controlling and blocking the transmission of vector-borne diseases [9]. This method is described as “the using of different symbiotic microorganisms for delivering molecules with anti-parasitic or anti-vector effects to the wild vector population” [2, 10–15].

Recently, different research studies have been performed on symbiont microorganisms such as viruses, fungi, yeasts and bacteria as paratransgenesis agents [14, 16–25]. Given the fact that bacteria are more available and simpler than other microorganisms, they have been at the focus of research for developing paratransgenesis tools against VBD. Therefore, numerous studies have been performed to reach this goal, and different bacteria have been introduced as suitable candidates for paratransgenesis, especially for combating malaria [2, 9, 10, 12, 13, 16, 20, 24, 26–39]. Among the introduced candidates, bacteria of the genus *Asaia* have special characteristics that differentiate them from the other introduced bacteria [11, 40, 41]. *Asaia* spp. belong to the acid acetic alpha proteobacteria and are stably associated with some mosquito species such as *Anopheles* spp., *Culex* spp. and *Aedes* spp. Thus, *Asaia* spp. have been the target of studies on vector microbiota [42–46]. *Asaia* spp. have some features that persuade researchers to consider these bacteria as appropriate candidates for paratransgenic control of malaria and other VBD. Cultivation in cell-free media, colonization in different parts of the vector’s body (gut, salivary gland, reproductive organs, etc.), simple transformation capacity, transstadial and transovarial transmission and cryo-preservation capability are the main specific features of the species of this genus that discriminates it from the other symbiont microorganisms [40, 41, 46, 47]. Recently, bacteria have been introduced and used as dsRNA producing agents and delivery vehicles to insects for triggering the RNAi pathway against the targeted genes [48–57]. Therefore, with regard to the unique features of *Asaia* spp., unsuitable environmental conditions of malaria endemic regions, drawbacks of the research-based dsRNA administration routes to insects and the costs of *in vitro* dsRNA production, these bacteria could be considered as valuable and cost-effective candidates for applying the RNAi technology against malaria and other VBD in endemic regions.

Nevertheless, there is an important point that should be considered for increasing the effectiveness of the developed method based on symbiont bacteria and RNAi technology. Studies have shown that the dsRNA-specific endonuclease (RNase III) mutant bacteria are more efficient for producing the dsRNA molecules in quantity in comparison with their wild strains [49, 54, 57–60].

In fact, dsRNA substrates are subjected to cleavage by a specific superfamily of dsRNA endonucleases that existed in most of prokaryotes and eukaryotes [61–65]. Ribonuclease III (RNase III), an endoribonuclease, is encoded by the *rnc* gene, is an Mg^2+^ dependent enzyme that is important for ribosomal RNA (rRNA) processing, cellular defense against the viral infections and post-transcriptional gene expression control [61, 63–67]. Hence, we decided to characterize the *rnc* locus and its related protein in a field-isolated *Asaia* sp. by genomics, proteomics and *in silico* approaches to provide the basic and essential information for developing an *rnc* mutant *Asaia* strain. Our final goal was to utilize the information to create a strain which could be used as a pluripotent and robust dsRNA production tool and delivery vehicle against target genes in *Anopheles* sp. (larvae and adults) to control malaria vectors.

## Methods

### Primer design

*16S* rRNA gene primers were used from our previous study [46, 67]. To characterize the *rnc* gene from the isolated *Asaia*, full characterized sequences of the *rnc* gene from different acetic acid bacteria species were aligned using MEGA v.7.0 software [68]. After alignment, nine conserved regions were selected for designing the five forward (F1, F2, F3, F4 and F5) and four reverse primers (R1, R2, R3 and R4) to determine the middle part of the sequence of the *rnc* gene from the isolated *Asaia* (Table 1). Thereafter, according to the middle part of the sequence, eight gene-specific primers (forward: F652, F624, F566 and F518; reverse: R32, R161, R182 and R245) were designed to carry out the 3ʹ- and 5ʹ-genome walking (Table 1).

**Table 1:** List of the primers designed for *rnc* operon characterization and *Asaia* sp. molecular identification

Primer name	Sequence (5ʹ–3ʹ)
16S rRNA-F	TGGCGGACGGGTGAGTATC
16s rRNA-R	AGTTGGTTTGACCCGAAGCC
F566-3W	CTTTACCATCGCGGTTTCTGCTATG
F624-3W	CGCCAGGCCGAGAGTGAG
F652-3W	CCCTGCTTCGGCGATTGAAC
R32-5W	TCGCAGAAGCCCCTGATCAG
R161-5W	CAGACCGAGCACACGATCAC
R182-5W	AAGCAGCCATTCAGCCATCAG
F1	GCCACCAGTTTGCTGATCAGG
F2	TGACGACTCATGACGCCATAGC
F3	CGTGTGCTCGGTCTGCTGATG
F4	CATATTGCGGAACATGAGGAGCAG
F5	GTTCAGGCCGACGCG
R1	ATGCCTTTACGGCCACGGTTCAATC
R2	GGCGGCGTGATCTGCGAG
R3	CCTCCATCGCGTCGGCCTG’
R4	CAGCCTCACTCTCGGCCTG
rnc-clF	AAGCTTGCATGACGACTCATGACGCCATAGCGGAG’
rnc-clR	CTCGAGTTTTTCTATCCTTCGTGCCTTTACGGCCAC

In the next step, rnc-clF and rnc-clR primers were designed to amplify and clone the coding sequence of the *rnc* gene in pET-28a expression vector (Table 1). All primers in this study were designed using the GeneRunner software (v.4.0.9.68 beta) and their specificity was evaluated by the nucleotide BLAST web-based software (https://blast.ncbi.nlm.nih.gov/Blast.cgi).

### Specimen collection and rearing

Adult and larval specimens of *Anopheles stephensi* were collected from Sistan and Baluchistan, Hormozgan and Fars provinces, in the southern regions of Iran. Eggs and larvae from the F1 generation of *An. stephensi* were first reared in the local insectaries, then sent to the Malaria and Vector Research Group (MVRG) insectary in Pasteur Institute of Iran, Tehran, where they were raised. On arrival, the larvae and adults were identified morphologically to species according to the Iranian *Anopheles* key [69] and identifications were confirmed with molecular amplification of the internal transcribed spacer 2 (ITS2) and the cytochrome *c* oxidase subunit 1 (*cox*1) gene for *An. stephensi*. Insectary conditions were designed to mimic the local field conditions: 60–80% humidity, a temperature range of 26–28 °C and a 12-h light/dark photocycle in near-axenic conditions during both the developmental and adult stages [70, 71].

### Isolation of *Asaia* sp. and molecular confirmation of the isolated bacteria

To prevent the probable surface contamination, all mosquitoes were initially immersed in 70% ethanol, rinsed by the sterile phosphate-buffered saline (PBS) and dissected separately. The larvae and adults were dissected under a steromicroscope under a biosafety level II biological laminar flow cabinet. The midgut of each sample was dissected, ground and then individually inoculated in *Asaia*-specific enrichment medium at 30 °C for three days with shaking at 180× *rpm* [72]. The ingredients of this medium were 2.0% d-sorbitol, 0.5% peptone, 0.3% yeast extract and 100 ppm cycloheximide at pH 3.5 with hydrochloric acid adjustment to provide specific conditions for *Asaia* sp. growth and isolation [72]. Subsequently, turbid tubes which indicated to the growth of bacteria were selected for plating on CaCO_3_ agar medium overnight to get the separated and morphologically defined colonies. This medium contained 2.0% d-glucose, 0.5% ethanol, 0.7% yeast extract, 0.8% CaCO_3_ and 1.2% agar at pH 8.6 [72]. In the next step, separated colonies were cultured in LB broth medium for preservation at – 80 °C and DNA extraction.

DNA was extracted from 1 ml of the overnight culture of bacteria in LB broth medium using the MBST genomic DNA extraction kit (MBST, Tehran, Iran) according to the manufacturer’s instruction. For molecular characterization and confirmation of the isolated colonies, AsaF and AsaR primers were used to amplify a 1200-bp amplicon of the *16S* rRNA gene (Table 1). All PCR reactions were performed in a total volume of 20 μl. The reaction mixture contained 400 nM of each primer, 1 unit of Taq DNA polymerase, 0.2 mM (of each) dNTPs, 2 µl of 10× reaction buffer, 1.5 mM MgCl_2_ and 150 ng of genomic DNA as template. The amplification profile was as follows: initial denaturation at 94 °C for 5 min; 35 cycles of 94 °C for 30 s, annealing at 62 °C for 30 s and extension at 72 °C for 1 min; with a final extension step at 72 °C for 10 min.

Amplified fragments were recovered using agarose gel extraction kit (GeneAll Biotechnology, Seoul, South Korea) according to the manufacturer’s instructions and TA-cloned into a pTG19-T cloning vector (Vivantis, Selangor, Malaysia). Plasmids of the positive colonies were purified using a plasmid extraction kit (GeneAll Biotechnology) and subjected to direct sequencing (Microgen, Seoul, South Korea) by universal primers. Sequencing results were analyzed and edited by Chromas v.2.31 and final alignment was performed by nucleotide BLAST web-based software for confirmation (https://blast.ncbi.nlm.nih.gov/Blast.cgi).

### Molecular characterization of the *rnc* gene sequence

Five forward and four reverse primers were designed for determining the middle part of the sequence of the *rnc* gene. Twenty PCR reactions were performed based on the possible combinations of F1, F2, F3, F4 and F5 as forward and R1, R2, R3 and R4 as reverse primers (Table 1). The amplification profile of the performed reactions was as follows: initial denaturation at 94 °C for 5 min; 35 cycles of 94 °C for 30 s, annealing at 62 °C for 30 s and extension at 72 °C for 50 s; with a final extension step at 72 °C for 10 min. The amplicon size of each reaction was estimated based on the genetically related species and finally, amplicons within the expected size ranges and higher length in comparison with the others were selected for TA-cloning and sequencing.

To achieve the full sequence of the *rnc* gene, the rapid amplification of genomic ends method was used. This step was performed based on our previous study [73]. In brief, for determining the 5ʹ-end of the *rnc* gene, single-stranded DNA (ssDNA) molecules were created by R182 as the outermost gene specific primer (with regard to the 5ʹ**-**end of the *rnc* gene middle part sequence) in the first step. Then, Genome Walking primers (GWPs) (A-G) were used for dsDNA production in seven separate reactions. Next, R162-UAP-N1 and R32-UAP-N2 primer pair combinations were used for the nest-1 and nest-2 reactions, respectively (Table 1). All PCR conditions and ingredients were the same as in our previous study [73]. In brief, ssDNA molecules were synthetized using R182 according to the following program: initial denaturation at 94 °C for 5 min; 25 cycles of 94 °C for 30 s, annealing at 65 °C for 30 s and extension at 72 °C for 3 min; with a final extension step at 72 °C for 10 min. Double stranded DNA molecules were synthetized using GWPs according to the following program: initial denaturation at 94 °C for 5 min; 35 cycles of 94 °C for 30 s, annealing at 28 °C for 30 s and extension at 72 °C for 3 min; with a final extension step at 72 °C for 10 min. Nest-1 and nest-2 reactions using the R162-UAP-N1 and R32-UAP-N2 primer pairs were performed with the following program: initial denaturation at 94 °C for 5 min; 35 cycles of 94 °C for 30 s, annealing at 60 °C for 30 s and extension at 72 °C for 3 min; with a final extension step at 72 °C for 10 min. Finally, the related amplicons of different GWPs within acceptable size range were selected for TA-cloning in pTG19-T (Vivantis) and sequencing.

The 3ʹ**-**end sequence determination was performed in the same way as the 5’**-**end sequence determination, except for the use of different specifically designed primers for this region. In brief, ssDNA molecules were synthesized by F652 primer as the outermost gene specific primer. As previously, GWPs (A-G) were used for dsDNA synthesis in seven separate reactions. Next, F624-UAP-N1 and F566-UAP-N2 primer pairs were used to perform two asymmetric nested-PCR reactions (Table 1). Selection of amplicons was performed as for the 5ʹ**-**end sequence determination and the selected amplicon were TA-cloned and sequenced.

In the next step, three parts of the *rnc* gene sequences (middle, 3ʹ**-**end and 5ʹ**-**end) were assembled using GeneStudio Pro software v.2.2.0.0 and the full-length coding sequence (CDS) of the *rnc* gene from the isolated *Asaia* was defined. Finally, rnc-clF and rnc-clR primers were designed to amplify this region for recombinant RNase III production in *Escherichia coli* (Table 1).

### Recombinant RNase III expression and its proteomics analysis

To express the RNAse III, CDS of *rnc* gene was amplified by the Expand High Fidelity PCR System (Roche, Penzberg, Germany) by rnc-clF and rnc-clR primers and TA-cloned into pTG19-T vector (Vivantis) in the first step. Then, the coding sequence was sub-cloned into the pET-28a by digestion with *Hind*III and *Xho*I restriction enzymes (Thermo Fisher Scientific, Massachusetts, USA). Finally, recombinant protein expression was performed into *E. coli* BL21 (DE3) strain by 0.4 mM IPTG induction for 15 h [70].

Pre-heated samples at 95 °C for 5 min were run at 90 mA of constant current for 90 min on 12% polyacrylamide gel (Bio-Rad, California, USA) and proteins were analyzed by Coomassie Brilliant Blue stain (Sigma-Aldrich, Missouri, USA). Electro-transfer to nitrocellulose sheets (Amersham, Little Chalfont, UK) was performed as described by Towbin et al. [74] with a trans-blot SD semi-dry transfer cell (Bio-Rad). After transferring, blocking was performed using bovine serum albumin (0.3 g BSA in 15 ml PBS at pH 7.5) at 4 °C overnight. The 6x-His tag rabbit polyclonal antibody (1:4000) (Abcam, Cambridge, UK) was used as the first and horseradish peroxidase-conjugated anti-rabbit antibody (1:5000) (Santa Cruz Biotechnology, Dallas, USA) was used as the second antibody to visualize antigen-antibody interactions. Visualization was done by incubating the membrane with SIGMAFast diaminobenzidine (DAB) with metal enhancer (Sigma-Aldrich). Purification of the recombinant protein was performed by Ni-NTA Agarose (Qiagen, Hilden, USA) in hybrid situation according to the Thermo Fisher Scientific protocol. Finally, quality of the purified recombinant protein was evaluated by SDS-PAGE and confirmed by western blotting as previously mentioned.

### Constructs for synthesis and production of dsRNA molecules

Considering the importance of carboxypeptidase-1 enzymes of *An. gambiae* and *An. stephensi* in the sexual development of *Plasmodium falciparum* [70, 75], the *cpbAs1* gene of *An. stephensi* was selected as the target gene for designing and producing dsRNA molecules to evaluate their effects on the target gene expression in the future experiments. This gene has three exons and two introns in *An. stephensi* and 450-bp from the end of the first exon was selected as the target region for the design of dsRNA. A 150-bp linker in length was used to connect two antiparallel sequences and *BamH*I and *Xho*I restriction sites were added to the 5ʹ- and 3ʹ-ends of the designed sequence respectively. This construct was synthesized by ShineGene (Shanghai, China) in pUC57 vector and then sub-cloned into the pET-28a expression vector by digestion with *BamH*I and *Xho*I restriction enzymes. At the end, dsRNA expression was performed in *E. coli* BL21 (DE3) strain by 0.4 mM of IPTG induction for 4 h; the produced dsRNA molecules were purified by TRIZOL Lysis Reagent (Sigma-Aldrich, Missouri, Germany) [76–78], recovered from the agarose gel and their quality and quantity were evaluated by agarose gel electrophoresis and Colibri Microvolume Spectrophotometer (Titertek Berthold, Bad Wildbad, Germany), respectively.

### Bioactivity analysis

In the first step, concentration of the purified RNase III enzyme was determined by Bradford assay [79]. To perform the bioactivity assay, 1 nmol of the purified dsRNA molecules was incubated with ~ 5 ng of the recombinant purified RNase III enzyme. The cleavage buffer, which contained 30 mM Tris (pH 7.5), 150 mM KCl, 5 mM spermidine, 20 mM MgCl_2_, 0.1 mM DTT and 0.1 mM EDTA, pH 7.5, was mixed with 1 nmol of dsRNA and incubated at 37 °C for 1 min. The reaction was initiated by adding 10 mM of MgCl_2_ at 37 °C and the degradation of dsRNA molecules was evaluated on 2% agarose gel electrophoresis after blocking the reaction by adding 20 mM EDTA in different time frames (0, 1, 2 and 4 min) [80–82]. In addition, the specificity of the recombinant protein was evaluated on the purified total RNA from the induced bacteria for dsRNA production.

### Phylogenetic analysis, modeling, refinement and quantitative evaluation of *Asaia* RNase III enzyme

After translation of the coding sequence of *rnc* gene with GeneRunner software v.4.0.9.68 beta, protein BLAST was performed for determining the highly similar proteins with our query sequence; proteins with high identity, coverage and score were selected to perform an alignment. Alignment was performed with Clustal Omega server based on the Clustal W [83] multiple sequence alignment method with some bacterial RNase III proteins such as *Aquifex aeolicus*, *Thermotoga maritima*, *E. coli*, *Shigella dysenteriae*, *Gluconobacter oxydans*, *Parasaccharibacter apium*, *Commensalibacter intestine*, *Acetobacter aceti* and *Komagataeibacter intermedius*, to analyze the conservation of the structurally important residues of RNase III enzyme superfamily in our target sequence [83]. Next, these sequences were analyzed with MEGA v.7.0 software based on the neighbor-joining method with 1000 bootstrap replicates [68].

Moreover, comparative homology modeling (HM) was performed to predict the three-dimensional structure of *Asaia* RNase III enzyme by using the SWISS-MODEL [84], Phyre2 servers [85] and Modeller v.9.19 software [86] and the best structures were selected according to the QMEAN (Qualitative Model Energy Analysis) and GMQE (global model quality estimation) [87–90]. SWISS-MODEL and Phyre2 are web-based integrated services for predicting and analysing protein structures, whereas Modeller is a computing system for comparative protein structure modeling. Comparative HM consists of four basic steps: template selection, template target alignment, model construction, and model evaluation and refinement [91]. Models were computed by the SWISS-MODEL and Phyre2 servers in an automated-normal mode that only required the amino acid sequence of the target protein as input. In parallel, based on the ranked discrete optimized potential energy (DOPE) score of 10,000 generated models in Modeller, the top ten predicted models were chosen for refinement and validation.

The stereochemical quality of the predicted protein structures from the both approaches was analyzed with several web tools to enhance the accuracy of evaluation. MolProbity [92], PROCHECK [93], VADAR [94], ERRAT [95], VERIFY_3D [96], ProSA [97] and PROSESS web tools [98] were used for structural analysis. The statistics of non-bonded interactions between the atoms in 3D structure in comparison with a database of the highly refined structures were evaluated by ERRAT and structures with higher scores reflected higher quality [95]. The compatibility of the 3D models with amino acid sequences was analyzed by Verify_3D. A 3D structure is considered as well compatible with its sequence when more than 80% of the amino acids are scored ≥ 0.2. Generally, the ProSA Z-score determines the overall model quality. Z-score plot displays whether the z-score of the input structure is within the range of scores that are typically found for native proteins with similar size. The structural similarity between the model and template was calculated by using the TM-align web server. The template modeling score (TM-score) is a measure of similarity between two protein structures with different 3D structures. Commonly, the structures with a score higher than 0.5 are assumed to have the same fold [99].

Furthermore, the best model was selected and considered for structure refinement by using the GalaxyRefine web server [100]. Then, suggested structure with the highest structural similarity was selected for superimposition and calculation of the root-mean-square deviation (RMSD) by using the Swiss-PdbViewer DeepView v.4.1.0 and UCSF Chimera v.1.11.2 [101].

### Molecular docking studies

The crystal structure of dsRNA was retrieved from the template crystal structure (PDB ID: 4M30) in the RCSB Protein Data Bank (PDB) and molecular docking experiments of the suggested structure by SWISS-MODEL was carried out with HADDOCK web server to understand the molecular interactions [102, 103].

### Promoter region and transcription termination mechanism analysis

BPROM, which is used for predicting the bacterial operon in immediately upstream regions of the ORF start site (prediction of bacterial promoters, Softberry Inc), was used for analyzing the promoter region of *rnc* gene based on the sigma 70 of RNA polymerase binding site [104]. Furthermore, ARNold and FindTerm (Softberry Inc.) web server software were used to predict the mechanism of transcription termination for *rnc* gene [105].

## Results

### *Asaia* sp. isolation from the field-collected mosquitoes

After inoculation by surface striking, uniformly smooth white colonies with and without dissolution halo were grown on CaCo3 agar medium. Different morphological colonies were selected for molecular analysis and confirmation. Finally, a smooth-white colony with dissolution halo from Hormozgan Province was selected for performing the next steps and further analysis.

### Molecular identification and confirmation of *Asaia* sp. based on the sequence of the *16S* rRNA gene

By performing PCR with *Asaia 16S* rRNA gene-specific primers, a 1200-bp amplicon was observed after agarose gel electrophoresis in all isolated colonies (with and without dissolution halo) which confirmed the species of isolated bacteria (Additional file 1: Figure S1). Sequence analysis by nucleotide BLAST confirmed their high similarity and identity to the reported sequences of *16S* rRNA gene of *Asaia* species in the GenBank. These sequences were submitted to the GenBank database under the accession numbers MK128664, MK616065, MK616094, MK616097, MK645713, MK645714, MK645722, MK645809, MK645810 and MK645851.

### Molecular characterization of the *rnc* gene

For middle part sequence determination, agarose gel electrophoresis revealed that between the 20 possible primer coupling situations, only combinations of F1 with all reverse primers (R1-R4), F4 with R3 and R4, F5 with R3 and R4 resulted in amplification of the expected amplicons. Because F1 and R1 were the outermost primers and their amplicon had more coverage of *rnc* gene in comparison with the other amplicons, this combination was selected for confirming with internal primers. Primer combinations F4-R3, F4-R4, F5-R3 and F5-R4 confirmed the relationship of the targeted amplicon to the *rnc* gene. Upon validation, the 685-bp amplicon generated with the F1-R1 primers was TA-cloned and sequenced. Nucleotide BLAST analysis revealed 99% coverage and 95% identity to the *Asaia bogorensis* genome (GenBank: AP014690.1). Finally, this part of the *rnc* gene from the isolated bacterium was submitted to GenBank (MG431206) and used to design the gene-specific primers (GSPs) for determining its upstream and downstream sequences by the genome walking method.

After performing the 3ʹ-genome walking, sequence analysis of the products of different DRTs (A-G) revealed the presence of overlapped regions at their 5ʹ-ends with 3ʹ-end of the middle part of the sequence. Therefore, the longest amplicon (925-bp) which was related to DRT-A was selected and submitted to GenBank (MK190424). The 5’-genome walking amplicons were analyzed likewise, and it was revealed that all of them had an overlapped region at their 3ʹ-end with the 5ʹ-end sequence of the middle part sequence of the *rnc* gene. The longest amplicon, which was related to DRT-C (897-bp), was submitted to GenBank (MK190423). The nucleotide BLAST analysis revealed its high similarity and homology (≥ 95%) with the *A. bogorensis* genome (AP014690.1).

Finally, the acquired sequences for the 5ʹ-end, middle part and 3ʹ-end of *rnc* gene were assembled with GeneStudio software and submitted to GenBank (MG431209). Different probable open reading frames (ORFs) of the assembled sequence were predicted by GeneRunner software and ORF related to RNase III enzyme was determined using protein BLAST. It was interesting that there were two ORFs other than *rnc* which subsequent analysis revealed were related to *era* and *lep* genes (MK190425 and MK190426). Nucleotide BLAST analysis of the coding sequence (CDS) of the *rnc* gene from the isolated bacterium revealed 100% coverage and 95% identity to the *Asaia bogorensis* genome with accession number AP014690.1.

### *rnc* gene characterization

**Fig. 1:**
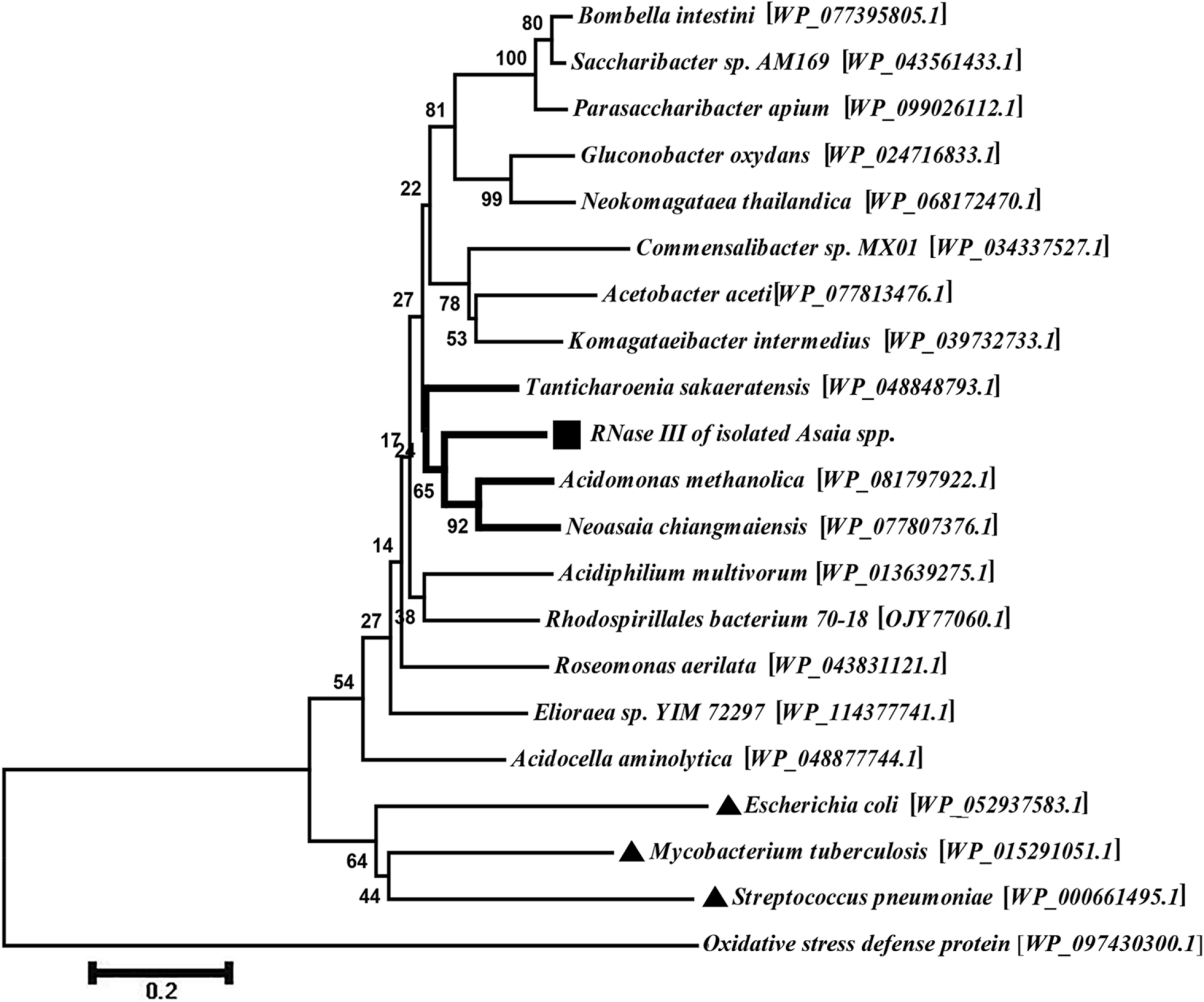
Phylogenetic tree for RNase III protein from the isolated *Asaia* sp. RNase III enzyme from the isolated *Asaia* sp. was aligned with the characterized RNase III enzymes from different bacteria and related species. This alignment and clustering was performed by MEGA7.0 software and neighbor-joining method with bootstrapping, respectively, to increase the accuracy of the created tree. The percentage of replicate trees in which the associated taxa clustered together in the bootstrap values (1000 replicates) is shown next to the branches. The scale-bar corresponds to 0.2 changes per residue. Our target protein is indicated with a black square and the branches of the more similar species are indicated by thick lines. Furthermore, subjects which have low similarity to others (*Escherichia coli*, *Mycobacterium tuberculosis* and *Streptococcus pneumoniae*) are marked by black triangles

Protein BLAST analysis of the coded protein by *rnc* gene showed that subjects with high identity and query coverage were related to the RNase III enzyme family of different bacteria species. Therefore, these were selected for alignment and phylogenetic tree analysis. Evolutionary analysis revealed that our target protein has a close relationship with those of *Acidomonas methanolica*, *Neoasaia chiangmaiensis* and *Tanticharoenia sakaeratensis*, all belonging to the family *Acetobacteraceae*. Moreover, our target protein has a more distant relationship with its counterparts in *E. coli*, *Mycobacterium tuberculosis* and *Streptococcus pneumoniae* (Fig. 1).

**Fig. 2:**
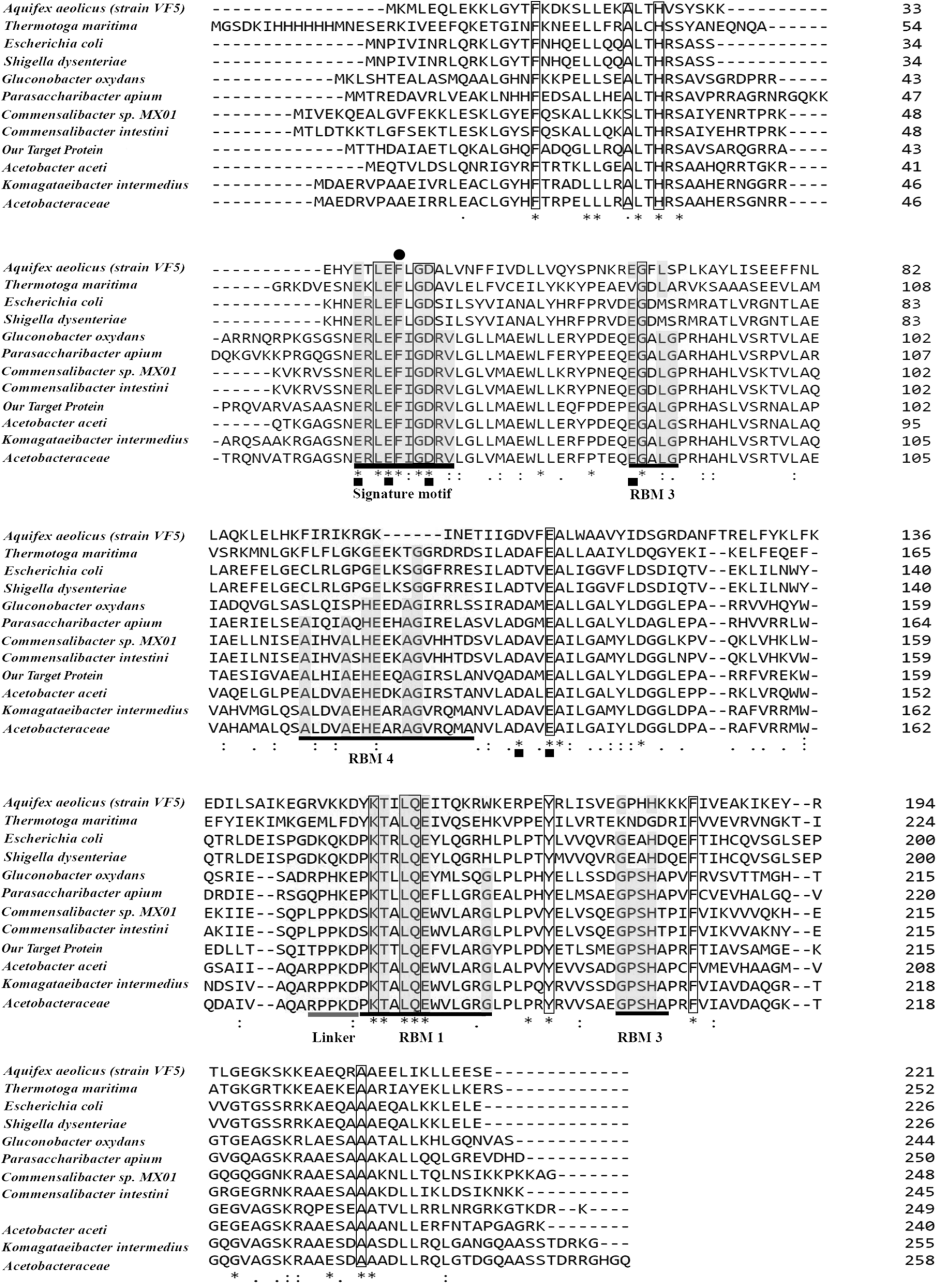
Conserved domain analysis of RNase III proteins with Clustal Omega. The alignment of our target protein with the structurally characterized bacterial RNase III was performed using the Clustal W multiple sequence alignment method. Conserved residues are indicated with gray rectangles; conserved residues between all characterized bacterial RNase III proteins are indicated with black rectangles. The signature motif consists of 9–10 residues that are almost conserved between all aligned sequences. RNA binding motifs (RBM1-4) and linker sequence are indicated by black lines. In addition, the important residues that are involved in active site and Phe61 which has an important role in dimerization of the enzyme are indicated by black squares and a black circle, respectively

According to the study by Court et al. [66] on *Aquifex aeolicus* RNase III structure, alignment of the amino acid sequences of similar RNase III enzymes by Clustal Omega revealed that the structurally important residues are conserved in all aligned sequences (Fig. 2). The structural signature of RNase III enzymes is a divalent metal ion (such as Mg^2+^, Mn^2+^, Co^2+^ and Ni^2+^) dependent domain which is crucial for their biological activity. This alignment revealed that the glutamic acids at positions 57, 60, 136 and 84, and aspartic acids at positions 64 and 133 are involved in the construction of the catalytic domain of our target enzyme. Moreover, these residues, that are coordinated with divalent metal ions, were conserved between all aligned sequences. By structural comparison with other characterized RNase III enzymes, the secondary structure of our target protein, its signatures and motifs were determined (Fig. 2).

Further analysis of the target sequence and two other ORFs revealed that two ORFs other than *rnc* encode Era and lepB proteins. Therefore, additional analyses were performed for determining the probable relationship between these coding sequences. Our results revealed that *era-* and *lep-*coding sequences are located downstream and upstream, respectively, of the *rnc-*coding sequence and code Era and LepB proteins. We found that the *rnc-* and *era-*coding sequences have four nucleotides which overlap with each other. It means that the start codon (ATG) of the *era* is before the stop codon (TGA) of the *rnc* gene. Further analysis revealed that there is an inefficient ribosome-binding site (-GGT-) in comparison with the consensus sequence of shine-dalgarno (-GGAG-) before the start codon of the *rnc* gene which is located at positions − 5 to − 8 from the start codon of the *rnc* gene. Moreover, our analysis revealed that the *lep* gene, which encodes the signal peptidase I, is located in the upstream region of the *rnc* gene.

### 3D structure prediction and superimposition

Eight 3D structures were presented by SWISS-MODEL and Phyre2 web servers based on homology modeling. According to QMEAN and GMQE, the best predicted structure with the highest score was related to *Aquifex aeolicus* RNase III enzyme (strain VF5) (PDB: 4M30). Therefore, it was chosen as the reference molecule for performing further and detailed structural analysis.

### Validation of the predicted structure for *Asaia* RNase III enzyme

The stereochemical quality of protein structures of the ten top predicted models of Modeller and eight presented 3D structures by SWISS-MODEL and Phyre2 web servers were analyzed by utilizing several web tools to enhance the accuracy of evaluation. The related results to the final models of *Asaia* RNase III are presented in Tables 2 and 3. The number of serious clashes which showed unfavorable all-atom steric overlaps by more than 0.4Å per 1000 atoms was considered as the MolProbity clash score [92]. Better models possess a lower number of clash scores and clash scores of the final model of SWISS-MODEL, Phyre2 and Modeller were 1.66, 3.01 and 1.72, respectively, which were acceptable. The Verify_3D score of the suggested model of SWISS-MODEL, Phyre2 and Modeller was 72.01%, 67.07% and 82.13%, respectively (Table 2).

**Table 2:** Validation of the three predicted 3D models of *Asaia* RNase III by MolProbity, VADAR, Verify_3D and ERRAT

*Asaia* RNase III model	MolProbity score^a^	VADAR Free energy of folding	Verify 3D (%)^b^	ERRAT (%)^b^	Ramachandran plot quality (%)				Overall quality^c^
					Most favored	Additionally allowed	Generously allowed	Disallowed	
Swiss-model	1.66	− 383.46	72.01	91.98	89.2	9.6	1.0	0.2	4.5
Phyre2	3.01	− 191.89	67.07	82.35	85.4	11.3	1.9	1.4	3.5
Modeller	1.72	− 391.65	82.13	100	93.9	4.2	1.4	0.5	6.5

^a^MolProbity score combines the clash score, rotamer and Ramachandran evaluations into a single score

^b^100 is the best, 0 is the worst

^c^10 is the best, 0 is the worst

**Table 3:** Validation of the three predicted 3D models of *Asaia* RNase III model with ProSA and TM-score

*Asaia* RNase III model	QMEAN	ProSA^a^	TM-score^b^			
		Z-score	4M30	Swiss-model	Phyre2	Modeller
Swiss-model	− 4.44	− 5.54	0.899	–	0.55	0.81
Phyre2	− 3.37	− 5.52	0.531	0.55	–	0.56
Modeller	− 3.83	− 5.64	0.7732	0.81	0.56	–

^a^PRoSA Z-score determines the overall model quality

^b^TM-score has the value in (0, 1]. Higher score indicates generally the same folding

**Fig. 3:**
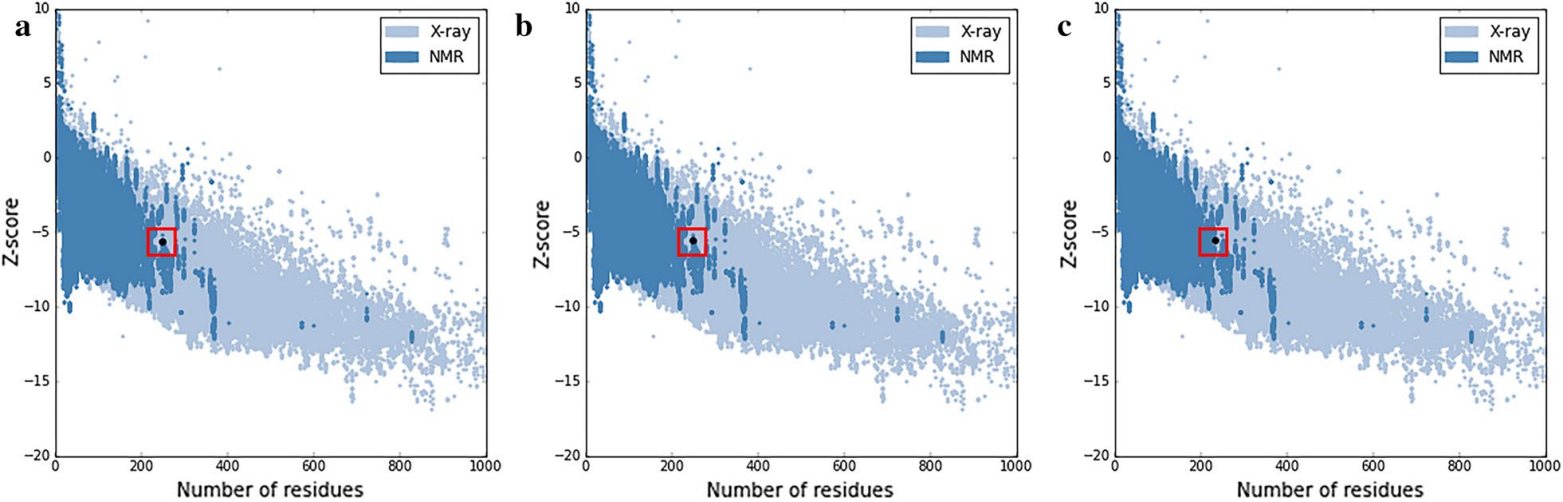
ProSA Z-score plots for final models of Modeller (**a**), Phyre2 (**b**) and Swiss-model (**c**). X-ray determined structures are colored light blue and NMR determined structures are colored dark blue. Large black dots show the ProSA Z-score of the predicted models by each method

Moreover, the structural results of ERRAT approved high overall quality of the final predicted 3D structures of SWISS-MODEL, Phyre2 and Modeller with the ERRAT scores which were 91.98%, 82.35% and 100%, respectively. The values for free energy of folding which was evaluated using the VADAR web server were − 383.46, − 191.89 and − 391.65 for the SWISS-MODEL, Phyre2 and Modeller predicted structure, respectively. Ramachandran plot results *via* PROCHECK showed that the stereochemical quality of the final selected models was satisfactory and an acceptable percentage of residues (89.2%, 85.4% and 93.9%, respectively) were located in the most favored region [93]. The overall quality of final models which was measured using the PROSESS web servers, gained scores of 4.5, 3.5 and 6.5 and admitted the overall quality of final models (Table 2). For three final models, ProSA Z-scores were located within the typical range of scores for the experimentally determined structures (Fig. 3).

The results of TM-score revealed that the predicted models by using the Swiss-model and Modeller have the same folding close to the template in comparison with the predicted model with the Phyre2 (Table 3). Therefore, the model generated by using the SWISS-MODEL was chosen for the docking study. The structure and sequence of the predicted models by SWISS-MODEL and Modeller were aligned with 4M30 as a template to determine the differences in the sequence that had led to the variation in folding patterns (Additional file 2: Figure S2).

Analysis of the final model by using the UCSF Chimera software revealed that RNase III of the isolated *Asaia* sp. bacterium is a globular protein with two important domains: (i) RNase III N-terminal catalytic domain (RIIID) and (ii) C-terminal dsRNA-binding domain (dsRBD) (Additional file 3: Figure S3a–c).

Based on the modeling and superimposition with the reference molecule, the catalytic center of our target protein consists of six acidic residues: four glutamic acids (E57, E60, E136 and E84) and two aspartic acids (D64 and D133), of which E57, E60 and D64 were located in the signature motif (Additional file 3: Figure S3d). In addition, it was revealed that there are four RNA-binding motifs (RBM1–4) in the predicted structure of RNase III enzyme, of which the 1st and 2nd were located in dsRBD and 3rd and 4th in RIIID motifs (Additional file 3: Figure S3e). Furthermore, it was found that each smaller domain (dsRBD) of the enzyme consists of 67 residues and are connected together by a short linker (five residues: 168–172) (Additional file 3: Figure S3f). Configuration and flexibly of the enzyme for identifying and biding to dsRNA molecules are dependent on the structure of this linker [66, 106, 107].

### Structural properties of the enzyme

Superimposition and comparison of critical residues of the active site revealed that the targeted residues are completely similar to their counterparts in *Aquifex aeolicus* RNase III and glutamic acid 60, aspartic acid 133, glutamic acid 136 and aspartic acid 64 are the base of catalytic domain that interact with divalent cations. These residues interact with a divalent cation in each monomer and catalytic domain of the enzyme which is constructed upon the dimerization (Additional file 4: Figure S4) [108].

**Fig. 4:**
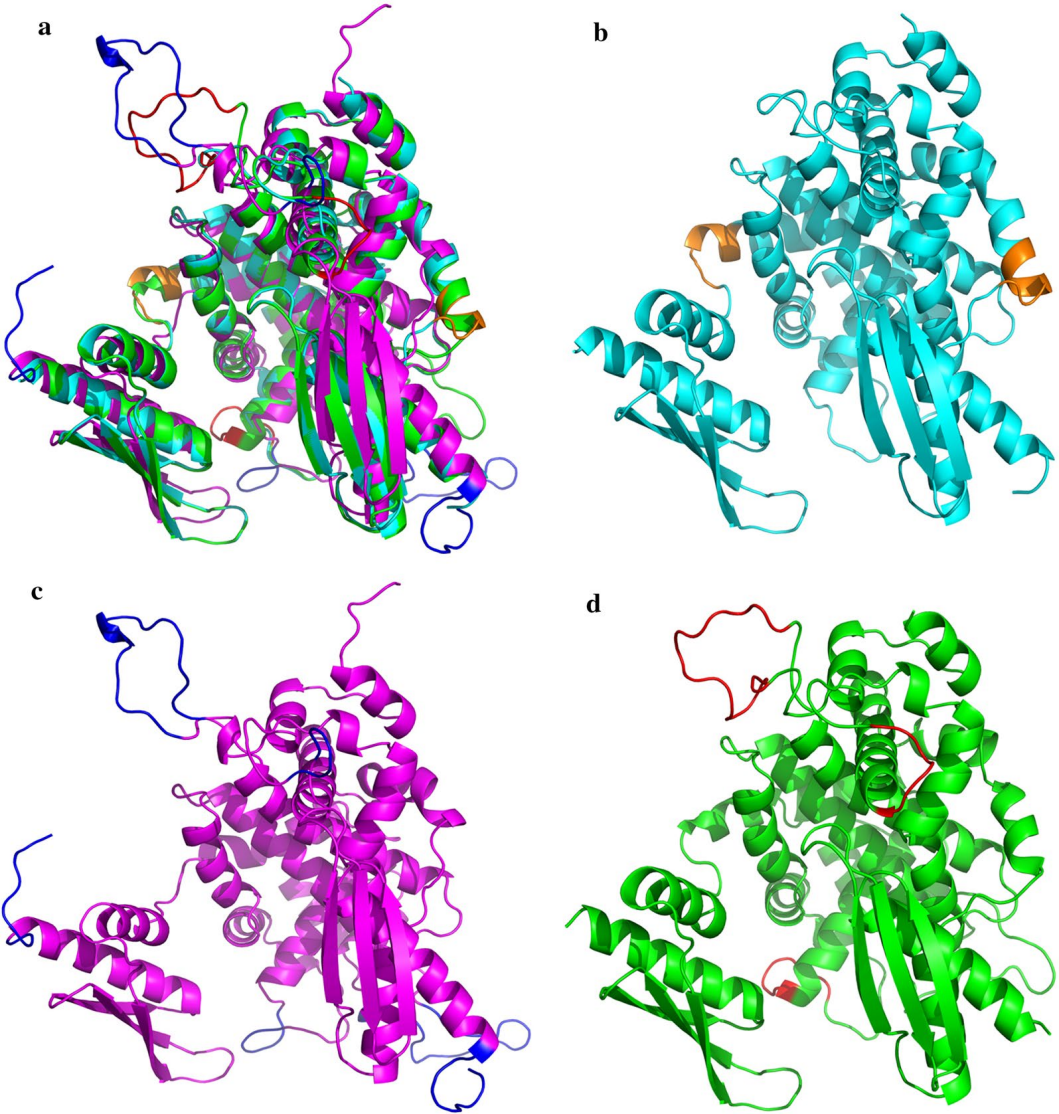
Structural alignment of predicted models and the crystal structure. **a** Superimposition of the 3D structure of 4M30 (light blue) with the predicted 3D structures of *Asaia* RNase III using the SWISS-MODEL (purple) and Modeller (green). **b** The 3D structure of 4M30 (light blue). **c** The predicted 3D structures of *Asaia* RNase III using the SWISS-MODEL (purple). **d** The predicted 3D structures of *Asaia* RNase III using the Modeller (green)

Comparison of their RMSDs with the reference molecule is presented in Table 4. This comparison revealed that the subtraction RMSDs values of the reference and target molecule at specific positions are less than 0.5 Å implying backbone similarity of the reference and target molecule (Fig. 4).

**Table 4:** RMSD values of the important domains of *Asaia* sp. RNase III in comparison to the *Aquifex aeolicus* RNase III

***Signature motif***										
Amino acid	**E**	**R**	**L**	**E**	**F**	**I**	**G**	**D**	**R**	**L**
Position	57	58	59	60	61	62	63	64	65	66
RSMD	0.322 Å	0.296 Å	0.166 Å	0.276 Å	0.420 Å	0.297 Å	0.325 Å	0.260 Å	0.118 Å	0.136 Å
***RBM 1***										
Amino acid	**P**	**K**	**T**	**T**	**L**	**Q**	**E**	**F**	**V**	**L**
Position	173	174	175	176	177	178	179	180	181	182
RSMD	0.292 Å	0.133 Å	0.262 Å	0.194 Å	0.174 Å	0.204 Å	0.200 Å	0.060 Å	0.204 Å	0.155 Å
Amino acid	**A**	**R**	**G**							
Position	183	184	185							
RSMD	0.200 Å	0.269 Å	0.700 Å							
***RBM 2***										
Amino acid	**G**	**P**	**S**	**H**	**A**	**E**	**G**	**A**	**L**	**G**
Position	198	199	200	201	202	84	85	86	87	88
RSMD	0.376 Å	0.526 Å	0.273 Å	0.532 Å	0.545 Å	0.151 Å	0.263 Å	0.163 Å	0.245 Å	0.151 Å
***RBM 3***										
Amino acid	**E**	**G**	**A**	**L**	**G**					
Position	84	85	86	87	88					
RSMD	0.151 Å	0.263 Å	0.163 Å	0.245 Å	0.151 Å					
***RBM 4***										
Amino acid	**A**	**L**	**H**	**I**	**A**	**E**	**H**	**E**	**E**	**Q**
Position	112	113	114	115	116	117	118	119	120	121
RSMD	0.293 Å	0.373 Å	0.468 Å	0.823 Å	0.519 Å	0.528 Å	0.736 Å	0.636 Å	2.201 Å	–
Amino acid	**A**	**G**	**I**	**R**	**S**	**L**	**A**			
Position	122	123	124	125	126	127	128			
RMSD	–	–	–	–	–	1.678 Å	0.528 Å

The values of overall RMSD between the final refined model and different ribonucleases were 2.369 Å for *Bacillus halodurans* RNase H (PDB: 1ZBF), 3.514 Å for *E. coli* RNase D (PDB: 1YT3), 2.845 Å for *E. coli* RNase E (PDB: 2C4R), 2.730 Å for *E. coli* RNase R (PDB: 1PCA), 1.266 Å for *Pseudomonas aeruginosa* RNase T (PDB: 2F96) and 3.886 Å for *Thermus thermophilus* RNase J (PDB: 3BK1).

### Molecular docking analysis

**Fig. 5:**
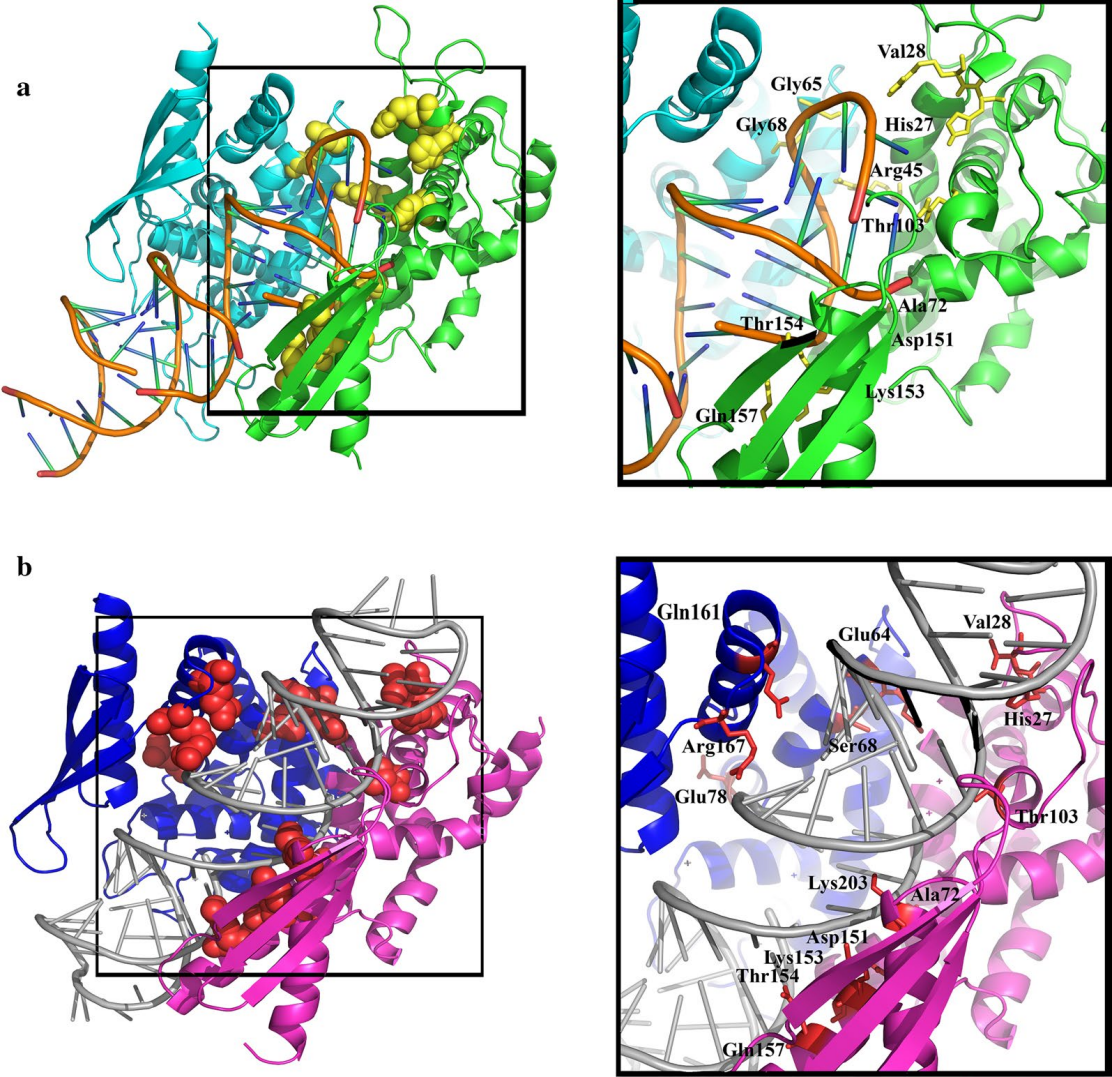
Interaction mode of the predicted model by Swiss-model and dsRNA in comparison with the 4M30. The complexes resulted from molecular docking which was conducted with HADDOCK. **a** Monomers of the predicted structure of RNase III are colored green and cyan. In close-up view, the residues of model that are involved in interaction with dsRNA are depicted in yellow and stick representation. **b** Binding model of dsRBD of *Asaia* RNase III dimer with dsRNA, the crystal structure of dsRBDs from each monomer is presented in blue and pink. In the close-up view, residues of protein that are involved in binding to dsRNA are illustrated in red and stick representation

Molecular docking studies could be helpful for figuring out the key residues and possible interacting faces in protein-protein interactions. Interestingly, although *Asaia* RNase III and *Aquifex aeolicus* RNase III share only 31% identity, patterns of their interactions with dsRNA were noticeably similar. Binding residues in RNase III (Dimer)-dsRNA interactions were investigated by LigPlot+ software [109] and are presented in Fig. 5.

### Prediction of promoter region and transcription termination mechanism

Analysis with BPROM software revealed the presence of a probable promoter sequence with at least 80% accuracy and specificity at position − 128 from the start codon of the *rnc* gene. Moreover, evaluation of the mechanism of transcription termination at about 1000-bp of the downstream of ORF sequence of *rnc* gene by ARNold and FindTerm showed that this phenomenon is not rho-dependent.

### Cloning, expression, purification and western blotting analysis of RNase III

**Fig. 6:**
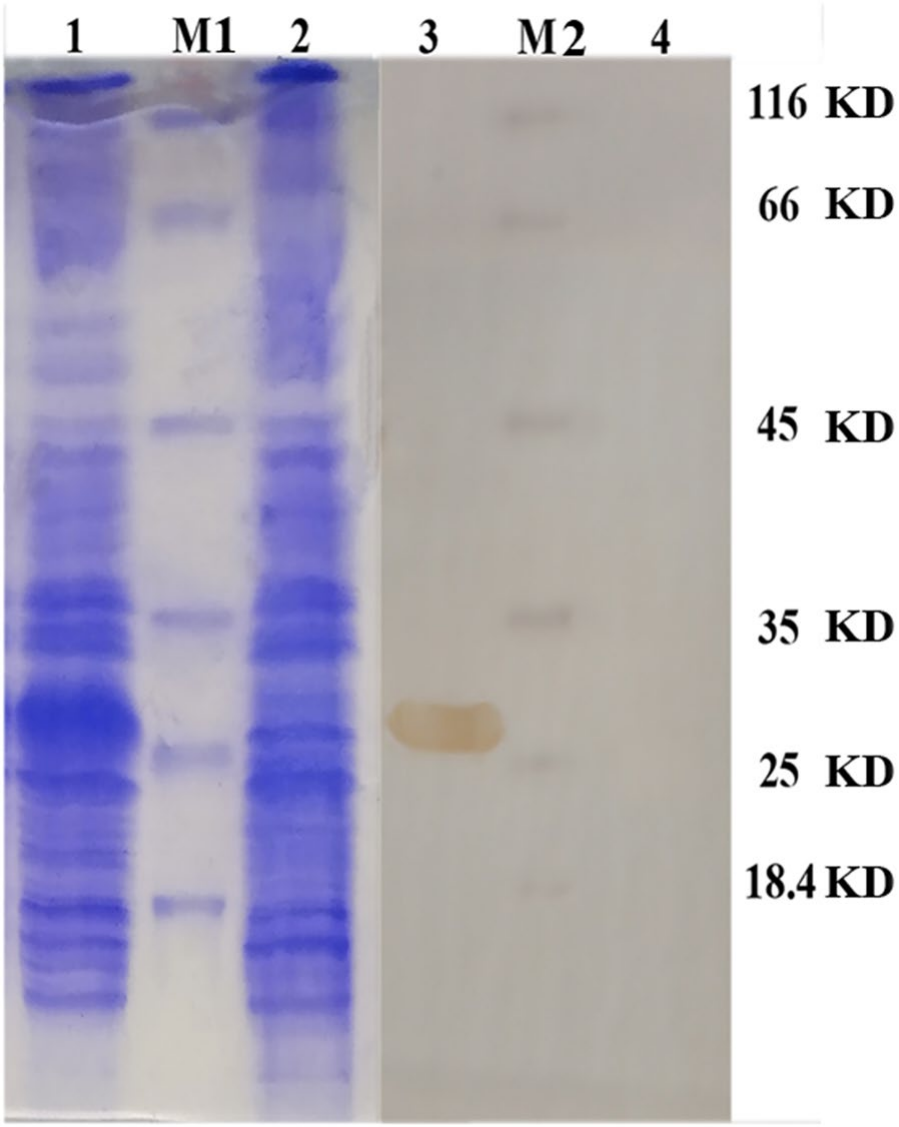
SDS-PAGE and western blot. After cloning in pET28-a and induction, the recombinant protein was analyzed by SDS-PAGE and western blotting assays. SDS-PAGE: Lane 1: clone with RNase III coding sequence, Lane M1: protein marker; Lane 2: clone without RNase III coding sequence. Western blot: Lane 3: clone with RNase III coding sequence; Lane M2: protein marker; Lane 4: clone without RNase III coding sequence

After overnight expression, a ~ 27.1 kD molecule was seen by SDS-PAGE analysis in comparison to the control that was confirmed by western blotting analysis based on the presence of 6XHis-tag fusion. The *Asaia* RNase III recombinant protein was purified by Ni-NTA agarose and the purification process was confirmed by SDS-PAGE and western blotting analysis (Fig. 6).

### dsRNA production

**Fig. 7:**
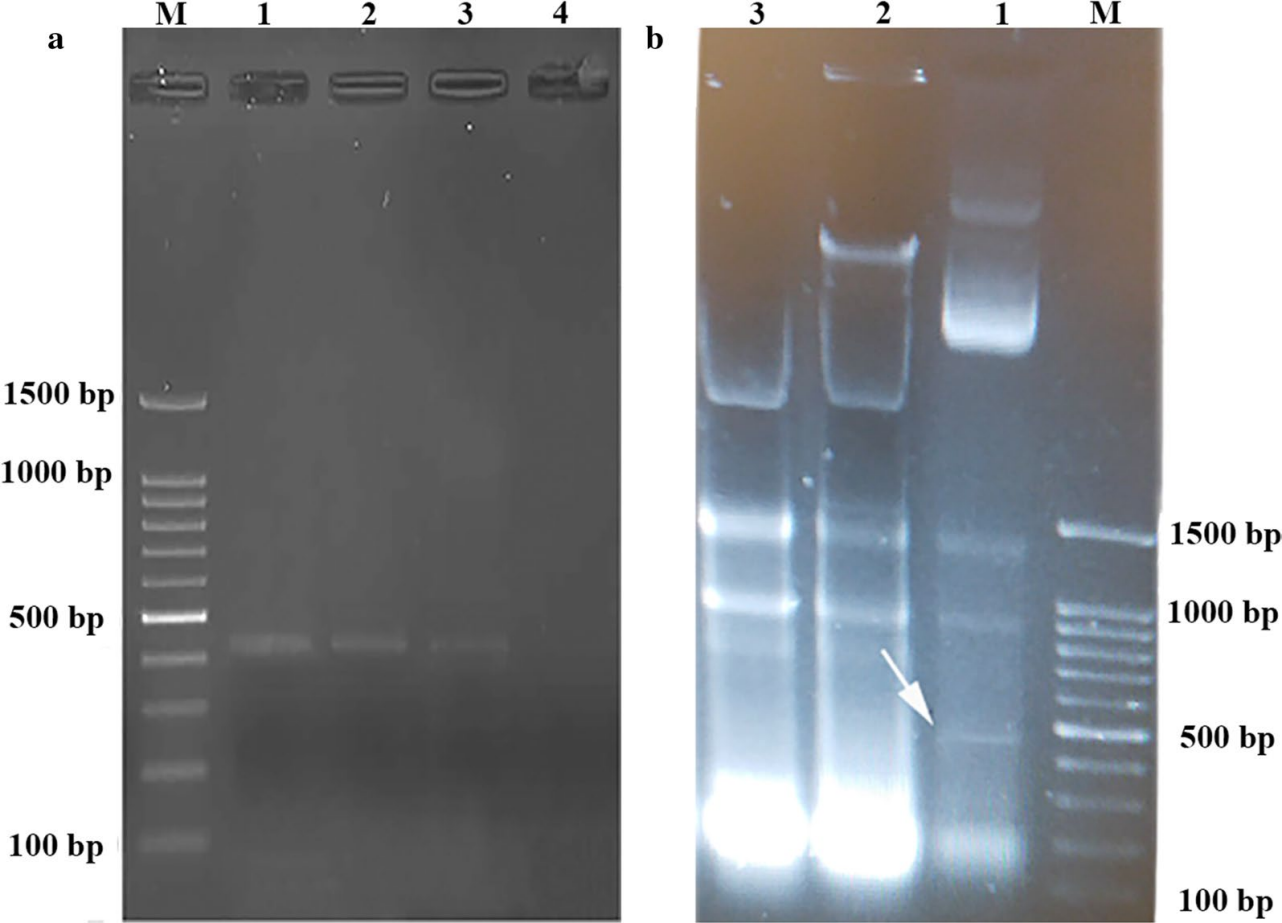
Biological activity of recombinant RNase III enzyme and specificity assay. **a** Purified dsRNA molecules were incubated with the purified RNase III recombinant protein for 1, 2 and 4 min. Lane M: marker; Lanes 1–4: 0, 1, 2 and 4 min after incubation, respectively. **b** For the specificity assay, purified total RNAs by QIAZOL from the transformed *E. coli* BL21 with dsRNA coding sequence plasmid were incubated with the purified recombinant RNase III. To perform this test, 1 nmol of the purified dsRNA molecules were incubated for 1 min with ~5 ng of the recombinant purified RNase III enzyme in appropriate buffer for RNase III enzyme activity. Lane M: DNA ladder; Lane 1: non-incubated sample with the recombinant *Asaia* RNase III protein; Lanes 2 and 3: incubated RNAs with the recombinant *Asaia* RNase III protein. The incubated samples were loaded on agarose gel three folds higher than the non-incubated sample for better visualization and analyses of dsRNA cleavaging. These analyses revealed that the recombinant *Asaia* RNase III protein degrades the dsRNA molecules specifically and has no effect on ssRNA molecules. The produced dsRNA is marked in Lane 1 with a white arrow

Agarose gel electrophoresis of the extracted total-RNAs from *E. coli* Bl21 (DE3) with and without dsRNA coding sequence after RNase A treatment revealed the presence of an amplicon about 500-bp in size in *E. coli* Bl21 (DE3) with dsRNA coding sequence that confirmed the production of our target dsRNA molecules (Fig. 7a).

### Enzymatic bioactivity

After stopping the dsRNA degradation assay reactions, the reaction mixtures were analyzed by agarose gel electrophoresis. The analysis showed that the purified recombinant protein degraded dsRNA molecules and its effect on dsRNAs was raised by increasing the incubation time (Fig. 7a). Furthermore, performing specificity analysis on the total purified RNAs revealed that it degraded dsRNA molecules specifically and had no effect on ssRNA molecules (Fig. 7b).

## Discussion

The double-stranded RNA endonucleases superfamily is divided to four classes: bacterial RNase IIIs, Rnt1p (in yeasts and fungi), Drosha and Dicer (in mammals) [66, 109–113]. RNase III (RNase III) is an Mg^2+^ dependent enzyme that is important for ribosomal RNA (rRNA) processing, cellular defense against the viral infections and post-transcriptional gene expression control [61, 63–66, 107]. For creating an RNase III mutant bacterium, it is necessary to have basic knowledge about the structure of the *rnc* gene, its surrounding sequences and structural properties of its coded protein for applying to the point mutations which result in the inhibition of its biological activity. Therefore, we characterized the *rnc* gene and evaluated the bioactivity and structure of its coded protein from an *Asaia* sp. which had been isolated from field-collected *An. stephensi* mosquitoes to provide the fundamental information for developing an efficient paratransgenic tool based on RNAi technology.

Coding sequence analysis of our target gene with the nucleotide BLAST server revealed that it is related to endoribonuclease III gene family and its sequence has considerable similarity with RNase III enzyme of different bacteria, especially in the *Acetobacter* family [66, 114–116]. According to the previous studies on other species of bacteria, *rnc*, *era* and *recO* genes are arranged as an operon: *rnc* is the first member of this operon and *era* and *recO* are located in the downstream region of *rnc* [117–120]. Our findings revealed that this arrangement is also seen in the isolated *Asaia* and that the *rnc-* and *era-*coding sequences had four nucleotides that overlapped, which is typical and similar to the previous studies on *E. coli*, *P. aeruginosa*, *Salmonella typhimurium* and *Rhodobacter capsulatus* [117, 121–123]. These findings are very critical for selecting an appropriate strategy for inactivating the RNase III without any side effects on the biological activity of other members of this operon. The presence of a weak Shine-Dalgarno sequence before the start codon of *rnc* gene is similar with the results of the study by March et al. on *E. coli* [117]. Moreover, this sequence has one of the shortest reported spaces between the ribosome binding site and the start codon in prokaryotes [117, 124]. It is concluded that this difference reflects the low level of cell demand for products of this operon [117, 118, 121].

Bacterial RNase IIIs consists of two distinct domains: RIIID and dsRBD [64, 66, 125]. The RIIID is composed of ~ 150 residues and includes 9–10 residues as the signature motif which are highly conserved in different species [66, 107, 116]. Based on homology modeling, the RIIID of our target protein is composed of 160 residues which include the signature motif and 16 residues with no counterparts in the reference model (4m30). These residues are seen as a protrusion on the surface of our target protein in the predicted model. Further analysis and alignment with the bacterial RNase III enzymes revealed that there is no equivalent for them in other bacterial RNase III enzymes except in the family *Acetobacteraceae*; it seems that this domain is specific to this family and has a particular function which should be further analyzed in future investigations. Comparison of our results with previous structural findings on RNase III enzymes of other bacteria revealed that the signature motif of our target protein consists of ten residues (57-ERLEFIGDRV-66) which are located in RIIID [66, 107, 114, 125]. In addition, based on the study by Blaszczyk et al. [111], Phe61 has an important role in the dimerization of enzymes, which is completely conserved among most bacterial RNase enzymes (Fig. 2).

Interaction with divalent metal ions is an important structural feature of RNase III enzymes which has been reported in different studies [66, 106, 111]. According to superimposition analysis, E60, D64 and E136 are involved in interaction with divalent metal ion in our target protein. Gan et al. [115] aligned 100 sequences of bacterial RNase III molecules and found that 15 residues (F19, A28, H31, L59, E60, G63, D64, G85, E136, K174, L177, Q178, Y191, F205 and A237) are conserved with high frequency in all of them. Molecular docking results of the predicted model by SWISS-MODEL with dsRNA were consistent with the findings of Gan et al. [115] and these residues were involved in Dimer-dsRNA complex conformation and their interactions. It was noticeable that all of these residues are conserved in the characterized bacterial RNase IIIs and our target protein (Fig. 2). Furthermore, we calculated the RMSD of these residues with the reference molecule and our results revealed that they have very close topology (Table 4).

The structure of RIIID is unique and is the signature of the RNase III family, but the structure of dsRBD might be seen in other dsRNA-binding proteins [66, 114]. dsRBD has an αβββα structure and there is only one copy of this structure in RNase III proteins, but there could be more than one copy in other dsRNA-binding proteins [66]. According to the *in silico* structural analysis, the dsRBD of our target protein has an αβββα structure similar to previous studies [64, 66, 67, 106, 107, 111, 125, 126].

## Conclusions

According to the fact that RNase III mutant bacteria are more efficient for producing the dsRNA molecules in comparison with the wild strains, characterization of the *rnc* gene and its related protein from an *Asaia* sp. was performed in this study. An important finding of our study was determination of the structural details of the *rnc* operon and its relationship with *era* and *recO* genes. Moreover, hot spot and structurally important residues in RNase III protein of *Asaia* sp. were determined by *in silico* analysis and docking approaches which is useful in paving the road of creating an *rnc* mutant *Asaia* strain; to the best of our knowledge, this study is the first report of the molecular characterization of an *rnc* gene and its related protein from *Asaia* species. At a glance, the data acquired in this study are informative for investigators that are interested in researching paratransgenesis, *Asaia* sp., and RNAi technology for combating malaria and other vector-borne diseases.

## Supplementary information

**Additional file 1: Figure S1.** Molecular confirmation of the isolated *Asaia* sp.. Molecular confirmation was performed based on *16S* rRNA gene amplification by the *Asaia*-specific primers. Lane M: DNA ladder; Lane 1: isolated bacterium; Lane 2: positive control; Lane 3: non-template control. The amplification of a 1200-bp amplicon confirmed that the isolated bacterium is *Asaia* sp.

**Additional file 2: Figure S2.** Sequence alignment of the predicted models and template by Clustal Omega. The sequence alignment of the predicted models by SWISS-MODEL, Modeller and 4M30 (as template) has been depicted to reveal the differences in the sequences that have an effect on the folding pattern. The conserved amino acids are shown in blue. Consensus residues are indicated by black rectangles.

**Additional file 3: Figure S3.** Three-dimensional structure of RNase III protein from the isolated *Asaia* sp. Three-dimensional structure prediction of our target protein was performed using SWISS MODEL and Phyre2 servers based on homology modeling. Our result shows that the RNase III from the isolated *Asaia* sp. is a globular protein with two distinct subunits which are connected together by a linker. **a**, **d** Front view of our target protein. **b**, **e** The complex of RNase III and dsRNA molecule. **c**, **f** Monomeric lateral view. N-terminal catalytic domains (RIIID), C-terminal dsRNA binding domain (dsRBD), dsRNA molecule, signature motif and liker are illustrated by purple, green, gray, yellow and red, respectively.

**Additional file 4: Figure S4.** The active site structure of *Asaia* sp. RNase III enzyme and its interaction with divalent cations. Six acidic residues are important and involved in catalytic domain construction and interaction with divalent cations. The specific residues have been determined and numbered and divalent ions are indicated with black circles. Their interactions are indicated by black lines.

## Data Availability

The sequences obtained and/or analyzed during the present study are deposited in the GenBank database under the following accession numbers: MK128664, MK616065, MK616094, MK616097, MK645713, MK645714, MK645722, MK645809, MK645810 and MK645851 for the *16S* rRNA gene; MK190424 for 3ʹ-genome walking; MK190423 for 5’-genome walking; MG431209 for the full length of the *rnc* gene; and MK190425 and MK190426 for *era* and *lep* genes. All other relevant data are included in the article and its additional files.

## Abbreviations

VBD: Vector-borne diseases
WHO: World Health Organization
MVRG: Malaria and Vector Research Group
ITS2: Internal transcribed spacer 2
*cox*1: cytochrome *c* oxidase subunit 1
PBS: phosphate-buffered saline
ssDNA: single-stranded DNA
GWPs: genome walking primers
CDS: coding sequence
HM: homology modeling
QMEAN: qualitative model energy analysis
GMQE: global model quality estimation
DOPE: discrete optimized potential energy
TM-score: template modeling score
RMSD: root-mean-square deviation
PDB: Protein Data Bank
GSPs: gene-specific primers
ORFs: open reading frames
dsRBD: dsRNA-binding domain
RBM: RNA-binding motifs
PII: Pasteur Institute of Iran
INSF: Iran National Science Foundation
BRC: Biotechnology Research Center
