# Supplementary figures and images for: Molecular characterization of RNase III protein of Asaia sp. for developing a robust RNAi-based paratransgensis tool to affect the sexual life-cycle of Plasmodium or Anopheles fitness
Source: Parasit Vectors. 2020 Jan 29;13:42. doi: 10.1186/s13071-020-3889-6 (PMC6990573; doi:10.1186/s13071-020-3889-6)

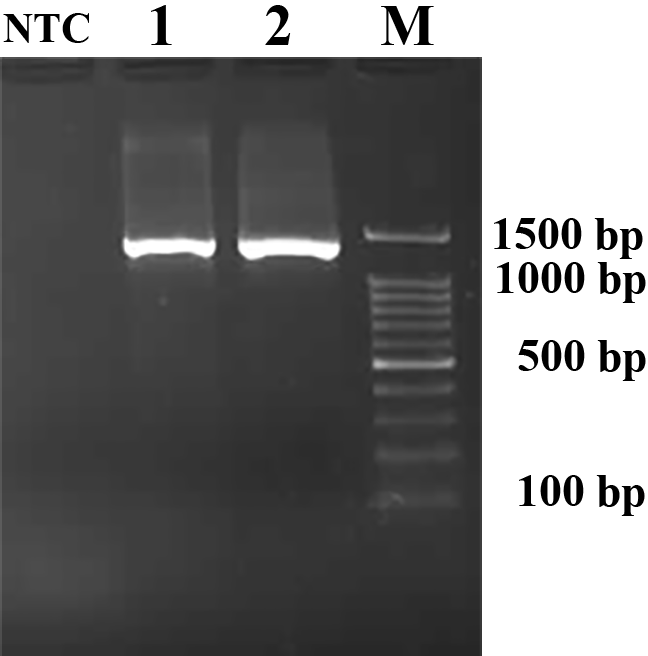

Supplement: Supplementary file 1 — Additional file 1: Figure S1. Molecular confirmation of the isolated Asaia sp.. Molecular confirmation was performed based on 16S rRNA gene amplification by the Asaia-specific primers. Lane M: DNA ladder; Lane 1: isolated bacterium; Lane 2: positive control; Lane 3: non-template control. The amplification of a 1200-bp amplicon confirmed that the isolated bacterium is Asaia sp. [file 13071_2020_3889_MOESM1_ESM.tif]

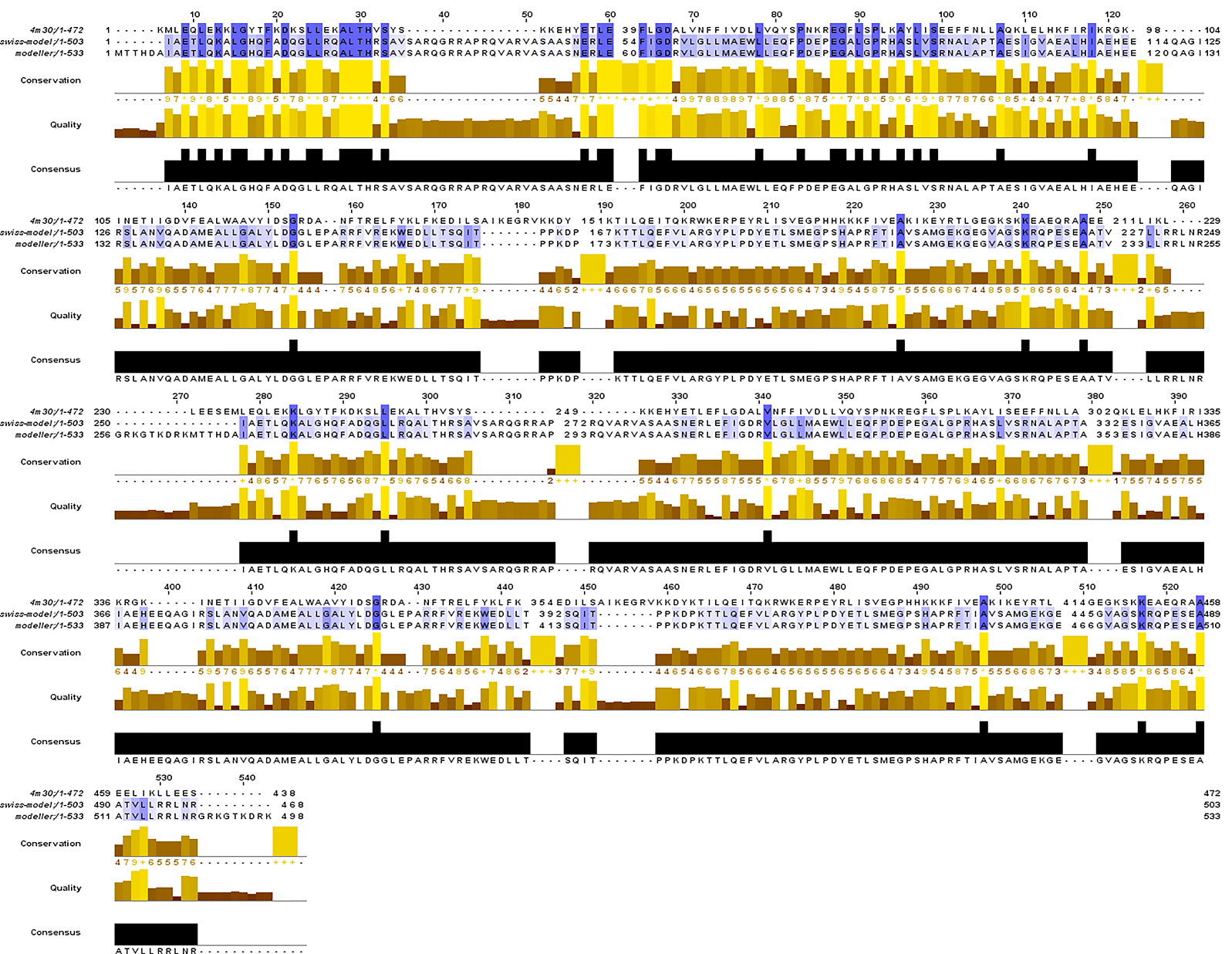

Supplement: Supplementary file 2 — Additional file 2: Figure S2. Sequence alignment of the predicted models and template by Clustal Omega. The sequence alignment of the predicted models by SWISS-MODEL, Modeller and 4M30 (as template) has been depicted to reveal the differences in the sequences that have an effect on the folding pattern. The conserved amino acids are shown in blue. Consensus residues are indicated by black rectangles. [file 13071_2020_3889_MOESM2_ESM.tif]

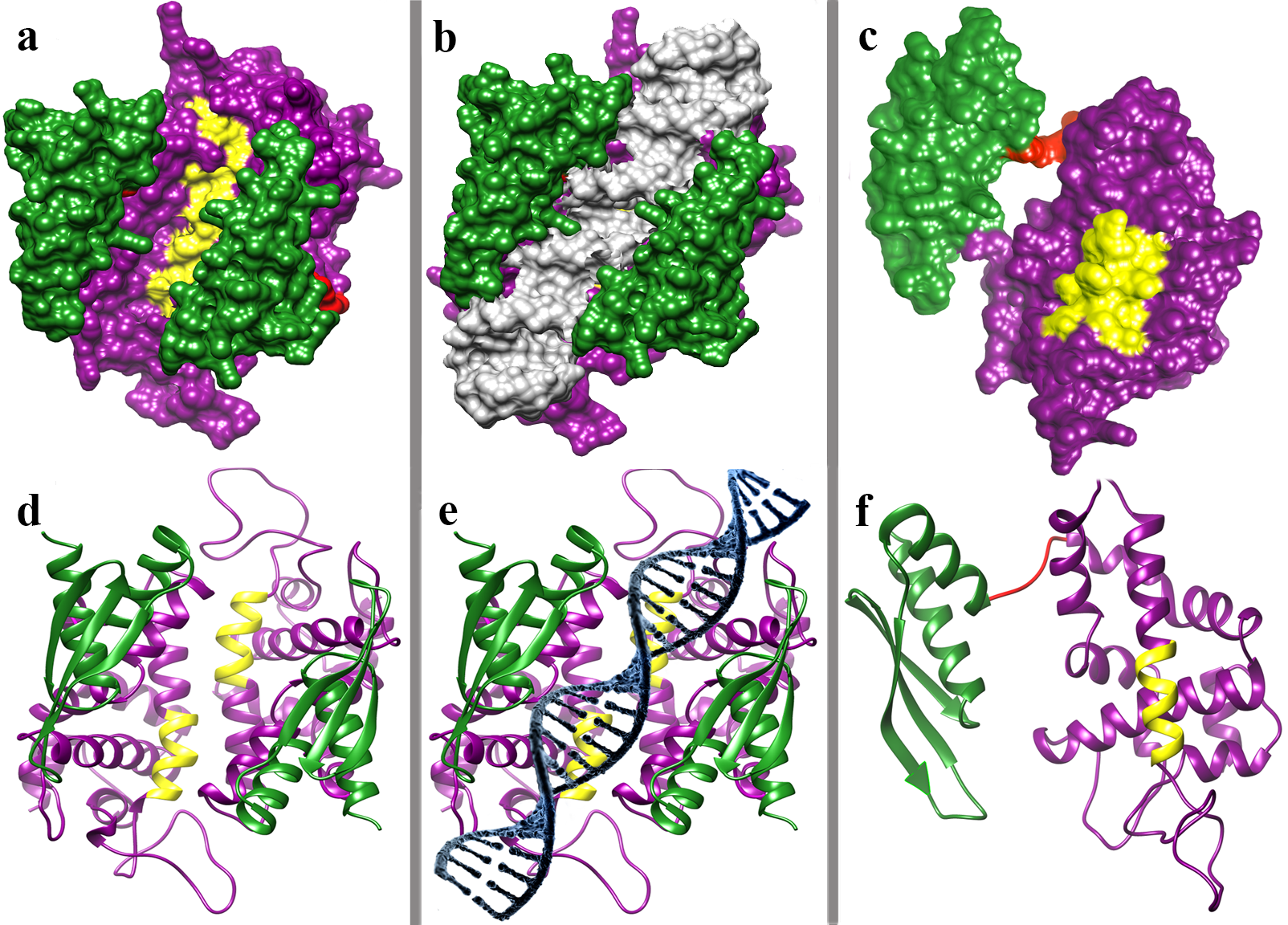

Supplement: Supplementary file 3 — Additional file 3: Figure S3. Three-dimensional structure of RNase III protein from the isolated Asaia sp. Three-dimensional structure prediction of our target protein was performed using SWISS MODEL and Phyre2 servers based on homology modeling. Our result shows that the RNase III from the isolated Asaia sp. is a globular protein with two distinct subunits which are connected together by a linker. a, d Front view of our target protein. b, e The complex of RNase III and dsRNA molecule. c, f Monomeric lateral view. N-terminal catalytic domains (RIIID), C-terminal dsRNA binding domain (dsRBD), dsRNA molecule, signature motif and liker are illustrated by purple, green, gray, yellow and red, respectively. [file 13071_2020_3889_MOESM3_ESM.tif]

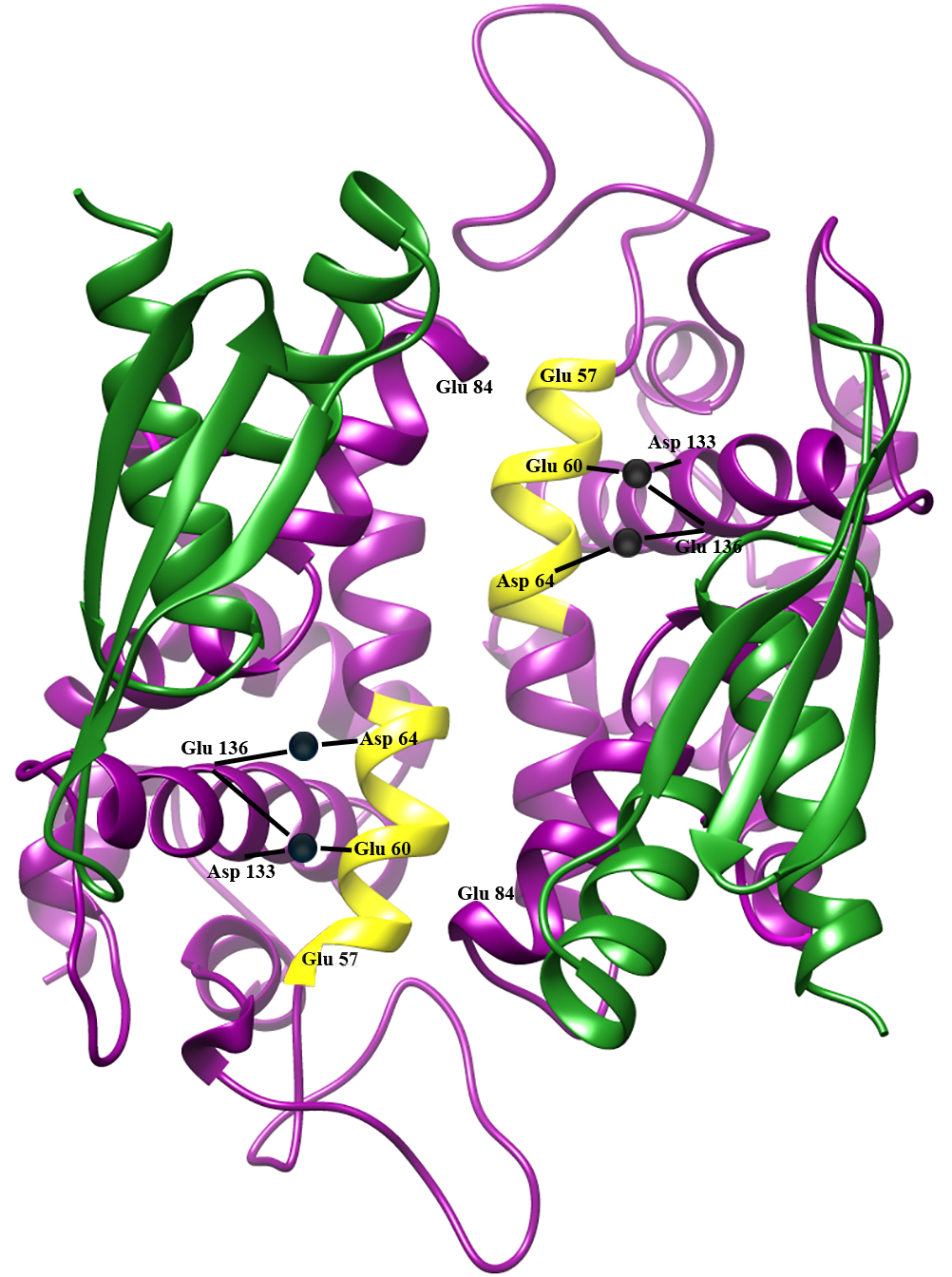

Supplement: Supplementary file 4 — Additional file 4: Figure S4. The active site structure of Asaia sp. RNase III enzyme and its interaction with divalent cations. Six acidic residues are important and involved in catalytic domain construction and interaction with divalent cations. The specific residues have been determined and numbered and divalent ions are indicated with black circles. Their interactions are indicated by black lines. [file 13071_2020_3889_MOESM4_ESM.tif]
